# ADAR1-dependent editing regulates human β cell transcriptome diversity during inflammation

**DOI:** 10.3389/fendo.2022.1058345

**Published:** 2022-11-28

**Authors:** Florian Szymczak, Roni Cohen-Fultheim, Sofia Thomaidou, Alexandra Coomans de Brachène, Angela Castela, Maikel Colli, Piero Marchetti, Erez Levanon, Decio Eizirik, Arnaud Zaldumbide

**Affiliations:** ^1^ ULB Center for Diabetes Research, Medical Faculty, Université Libre de Bruxelles, Brussels, Belgium; ^2^ Institute of Nanotechnology and Advanced Materials, Bar-Ilan University, Ramat Gan, Israel; ^3^ Department of Cell and Chemical Biology, Leiden University Medical Center, Leiden, Netherlands; ^4^ Department of Clinical and Experimental Medicine, University of Pisa, Pisa, Italy

**Keywords:** beta cell (β cell), inflammation, T1D (type 1 diabetes), transcriptome, RNA editing

## Abstract

**Introduction:**

Enterovirus infection has long been suspected as a possible trigger for type 1 diabetes. Upon infection, viral double-stranded RNA (dsRNA) is recognized by membrane and cytosolic sensors that orchestrate type I interferon signaling and the recruitment of innate immune cells to the pancreatic islets. In this context, adenosine deaminase acting on RNA 1 (ADAR1) editing plays an important role in dampening the immune response by inducing adenosine mispairing, destabilizing the RNA duplexes and thus preventing excessive immune activation.

**Methods:**

Using high-throughput RNA sequencing data from human islets and EndoC-βH1 cells exposed to IFNα or IFNγ/IL1β, we evaluated the role of ADAR1 in human pancreatic β cells and determined the impact of the type 1 diabetes pathophysiological environment on ADAR1-dependent RNA editing.

**Results:**

We show that both IFNα and IFNγ/IL1β stimulation promote ADAR1 expression and increase the A-to-I RNA editing of Alu-Containing mRNAs in EndoC-βH1 cells as well as in primary human islets.

**Discussion:**

We demonstrate that ADAR1 overexpression inhibits type I interferon response signaling, while ADAR1 silencing potentiates IFNα effects. In addition, ADAR1 overexpression triggers the generation of alternatively spliced mRNAs, highlighting a novel role for ADAR1 as a regulator of the β cell transcriptome under inflammatory conditions.

## Introduction

The type I interferon (IFN) response has recently been identified as a common signature for the development of autoimmunity (1). Induction of type I IFN (IFNα/β) following viral infection or endogenous release of mitochondrial genetic material is a highly regulated process in which pattern recognition receptors (PRR), such as MDA5, RIG-I and TLR3, act in concert to control inflammasome activation and the production of IFNα and IFNβ (2). In addition to this well-described sensing machinery, the adenosine-to-inosine conversion (A-to-I) catalyzed by the adenosine deaminase acting on RNA 1 (ADAR1) plays an important role in fine-tuning the innate immune response by destabilizing double-stranded RNA (dsRNA) duplexes and therefore reducing PRR substrate to limit further and potentially excessive inflammation (3).

ADAR1 exists as two isoforms that contain a central dsRNA binding domain and an enzymatic deaminase domain located in the C-terminal region. Both isoforms differ in localization (p110 remains mainly nuclear while p150 is expressed in the nucleus and cytosol) and by the presence of a nuclear export signal located in the N-terminus (4). ADAR1 has an essential role in modifying self-dsRNA formed by repetitive inverted elements, such as *Alu* short interspersed nuclear elements (SINE) elements, which inhibits the immune response triggered by the recognition of self-dsRNA by PRR.

Dysregulation of ADAR1 has been implicated in several interferonopathies, autoimmune diseases and tumor progression. Mutations within the RNA binding domain of ADAR1 alter both substrate affinity and specificity which affect RNA deamination and trigger the constitutive type I IFN response in Aicardi-Goutières syndrome (5). In contrast, high ADAR1 expression level has also been correlated with high tumor T-cell infiltrating lymphocytes (TIL) in breast cancer, and an increased amino acid substitution in the recognized antigens (a consequence of cytosine-to-uracil or adenosine-to -inosine editing at the RNA level) (6), demonstrating for the first time a role for RNA editing enzymes in the generation of tumor-specific neoantigens. Similarly, such processes have been proposed as a potential source of neoantigens involved in the development of autoimmune systemic lupus erythematosus (7).

In type 1 diabetes (T1D), increasing evidence indicate that local inflammation or other forms of stress combined with genetic predisposition leads to the generation and accumulation of aberrant or modified proteins to which central tolerance is lacking (8, 9). Examples of enzymatic deamidation or citrullination of self-antigens (e.g., proinsulin, C-peptide, GAD65, IA-2, GRP78, IAPP), as a consequence of activation of peptidyl arginine deiminase (PAD) or tissue transglutaminase (tTG) detected in pancreatic β cells in response to stress or primary islets from T1D patients, illustrate how the islet microenvironment can drive autoimmunity (10, 11).

RNA editing is a post-translational modification mediated by adenosine and cytosine deaminases which catalyzes the edition of a nucleotide into another in the context of an “editosome” (12). In addition to an amino acid change, RNA editing may enhance transcriptome complexity/diversity by directly changing splicing acceptor site motifs or altering splicing enhancer sequences with possible consequences for β cell immunogenicity (13, 14). In T1D, circulating T cells directed against alternative splice variants of GAD65, secretogranin V, CCNI-008, IAPP and Phogrin have been recently detected in patient blood samples and in the pancreatic islets (15).

To investigate the effect of the T1D pathophysiological inflammatory milieu on ADAR1 and the β cell transcriptome, we have analyzed high-throughput RNA sequencing data from human islets and EndoC-βH1 cells exposed to IFNα or IFNγ/IL1β, cytokines that contribute to the pathogenesis of T1D (16, 17). We demonstrate herein that inflammatory-mediated changes characteristic of early and late T1D development can trigger an increased A-to-I Alu editing rate. In addition, we demonstrate that ADAR1 not only dampens the innate immune response in β cells but also contributes to the transcriptome complexity with possible consequences for β cell function.

## Materials and methods

### Cell culture and treatment

EndoC-βH1 cells, kindly provided by Dr. Raphael Scharfmann (Paris Descartes University, France) (18), were maintained in low glucose DMEM supplemented with 5.5 μg/ml human transferrin, 10 mM Nicotinamide, 6.7 ng/ml Selenite, 50 μM β-mercaptoethanol, 2% human albumin, 100 units/ml penicillin and 100 μg/ml streptomycin. Cells were seeded in extracellular matrix, fibronectin pre-coated culture plates.

### Preprocessing and alignment of RNA sequencing data for the editome analysis

Raw FASTQ quality was assessed using FastQC version 0.11.8 and PCR duplicates were removed with Super Deduper (19). Remaining reads were uniquely aligned to the reference genome (hg38 and mm10 assemblies) using STAR (20) version 2.7.3a with parameters –alignSJoverhangMin 8 –alignIntronMax 1000000 –alignMatesGapMax 600000 –outFilterMismatchNoverReadLmax 1 –outFilterMultimapNmax 1.

#### Alu editing index

RNA Editing Index (21) version 1.0 was used to assess the overall editing in *Alu* elements, respectively. This measure calculates the average editing level across all adenosines in repetitive elements weighted by their expression, thereby quantifying the ratio of A-to-G mismatches over the total number of nucleotides aligned to repeats and comprising a global, robust measure of A-to-I RNA editing.

#### Quantification of expression

Abundance quantification was done using the quasi-mapping-based mode Salmon (21) (version 0.11.2) for human genome assembly hg38 with GENCODE version 24 and mouse genome assembly mm10 with GENCODE version 20. Gene expression analysis was later completed by using the tximport R package (version 1.12.3) to transfer Salmon’s isoform-level abundances to gene-level abundances (22, 23).

### RNA-sequencing and differential expression analysis

Total RNA was purified from EndoC-βH1 and EndoC-βH1/ADAR1 cells using the Nucleospin miRNA Kit (Bioke) according to the manufacturer’s guidelines. RNA quality was determined by Experion RNA StdSens 1K Analysis Kit (Bio-Rad, product number 7007103) on a Experion Automated Electrophoresis System (Bio-Rad) following the manufacturer’s protocol. Strand-specific bulk RNA sequencing was performed on a NovaSeq 6000 (2x150 paired-end with a depth of >150 million reads) by Eurofins Genomics Europe Sequencing GmbH (Konstanz, Germany). Reads were quality checked with fastp (24) to exclude reads of poor quality and remove remaining adapters. We used Salmon v1.3 (25) with additional parameters “–seqBias –gcBias –validateMappings” to quantify the gene and transcript expression. GENCODE was used as the reference genome and was indexed with default parameters. Differential Gene Expression (DGE) was performed with DESEq2 v.1.30.1 (26) with paired experiments included in the general linear model (i.e., ~ pairing + overexpression). For each gene, we obtained a log_2_ fold-change (log_2_FC), associated to an adjusted P value, which highlights the difference in gene expression between ADAR1-overexpressing cells and control cells. Gene Set Enrichment Analysis (GSEA) was performed using the above-generated DGE data with fGSEA (27). We pre-ranked genes according to the value of the Wald-test statistics provided by the DESeq2 output. Up- (enrichment) and down-regulated (depletion) pathways were considered significant when adj. P value < 0.05, regardless of their normalized enrichment score (**Supplementary Data S1**).

Gene-sets affected by alternative splicing were evaluated with clusterProfiler (28) with Gene Ontology as the reference (**Supplementary Data S2**).

### RT-PCR

Total RNA was extracted from EndoC-βH1 using NucleoSpin Kit (#740609.50S, Bioke). Approximately 0.5ug of RNA was used for reverse transcription. Oligo (dT) primers were used in the reaction. For siRNA experiments, RNA was isolated using Dynabeads mRNA DIRECT purification kit (Invitrogen, Carlsbad, California, USA) and reverse transcribed using Reverse Transcriptase Core kit (Eurogentec, Liège, Belgium). Real-time PCR amplification was done with SsoAdvanced Universal SYBR Green Supermix (BIO-RAD, Hercules, California, USA) and amplicons were quantified using a standard curve. Expression of the transcript of interest was detected using the following primers: ADAR1-p150 Fw GAATCCGCGGCAGGGGTAT, ADAR1-p150 Rv: GCTTAAGCAGGAAACTACTGGG; ADAR1 endogenous Fw: CCGCACTGGCAGTCTCCGGGTG, ADAR1 endogenous Rv: CCTGGCCCAGGCTGCTGGTACC, INS Fw: AAGAGGCCATCAAGCAGATCA, INS Rv: CAGGAGGCGCATCCACA, PDX1 Fw: CCATGGATGAAGTCTACCAAAGCT PDX1 Rv: CGTGAGATGTACTTGTTGAATAGGAACT, MAFA Fw: AGTCCTGCCGCTTCAAG, MAFA Rv: ACAGGTCCCGCTCTTTGG, NKX6.1 Fw: CTGGCCTGTACCCCTCATCA, NKX6.1 Rv: CTTCCCGTCTTTGTCCAACAA, GAPDH Fw: CCTGTTCGACAGTCAGCCG, GAPDH Rv: CGACCAAATCCGTTGACTCC; ACTIN Fw: CTGTACGCCAACACAGTGCT; ACTIN Rv: GCTCAGGAGGAGCAATGATC; STAT1 Fw: CACAAGGTGGCAGGATGTCT, STAT1 Rv: TCCCCGACTGAGCCTGATTA; MDA5 Fw: GTTGCTCACAGTGGTTCAGG; MDA5 Rv: GCTTGGCAATATTTCTCTTGGT; IFNB Fw: AGGACAGGATGAACTTTGAC; IFNb Rv: TGATAGACATTAGCCAGGAG; IFIT1 fw: CAGAATAGCCAGATCTCAGAGG; IFIT1 Rv: CCAGACTATCCTTGACCTGATG; CXCL10 Fw: AGTGGCATTCAAGGAGTACC; CXCL10 Rv: TGATGGCCTTCGATTCTGGA

### Western blot analyses

**Figure 1:**
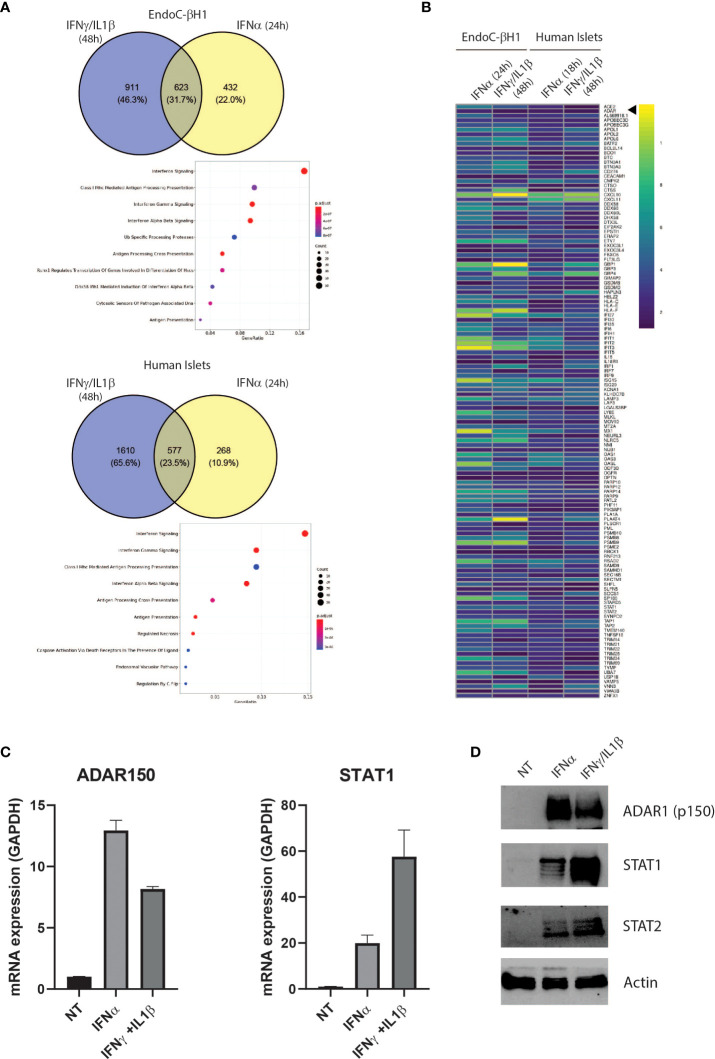
The proinflammatory cytokines IFNα and IFNɣ+IL-1β induce a partially shared gene signature in EndoC-βH1 and human islets Venn diagrams of up-regulated (log_2_FC > 0.58 and adj. P value < 0.05) genes in EndoC-βH1 and human islets after exposure to IFNɣ+IL-1β **(A)**, top) and IFNα **(A)**, bottom). Common genes have been tested for enrichment – using REACTOME as the reference – and significantly enriched pathways are represented as a dot plot: the x-axis represents the gene ratio and the y-axis the enriched pathways. **(B)** Heatmap representing the log_2_ fold change of the 128 up-regulated genes in all 4 datasets (|log_2_FC| > 0.58 and adj. P value < 0.05). **(C)** ADAR1 p150 and STAT1 gene expression in EndoC-βH1 were assessed by qPCR after cytokine treatment. n=3 independent experiments **(D)** ADAR1 p150 ADAR1 (detected using Anti-ADAR1 #ab168809, Abcam), STAT1 and STAT2 protein expression determined by western blot. β-actin expression was used as loading control.

**Figure 2:**
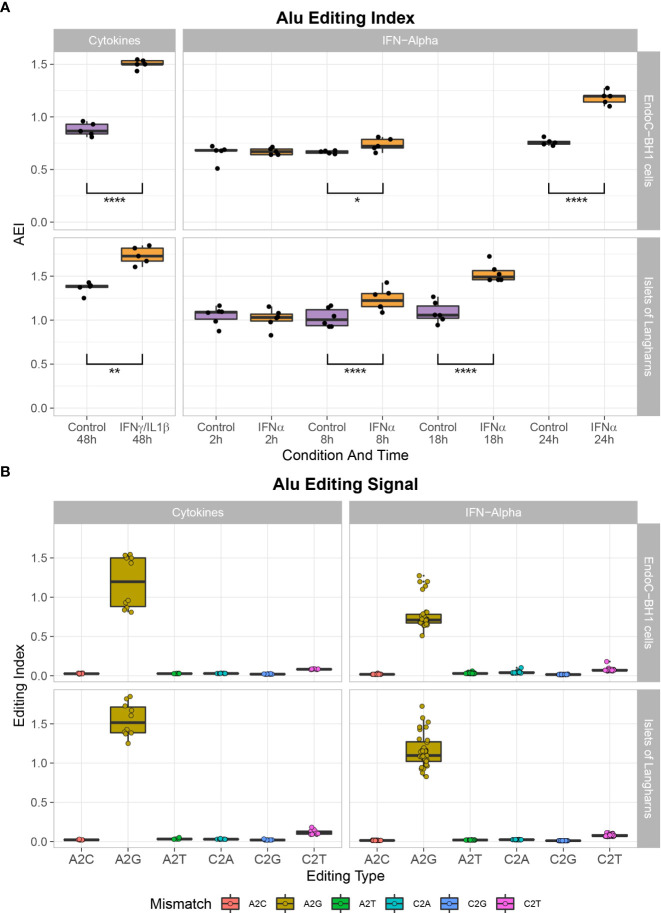
Cytokine treatment leads to A- to I- mutation in EndoC-βH1 and primary human islets. **(A)** Global A-to-I RNA editing index across *Alu* elements (short interspersed nuclear elements) in RNA-seq data demonstrates a higher A-to-I editing signal in IFNα or IFNɣ/IL1β stimulated samples after 8 hours and 48 hours, respectively. Student’s paired two-tailed t-test; *P<0.05, **P<0.005, ****P<0.0001. **(B)** Noise levels (non-A-to-G mismatches) are notably lower than seen in the global editing index’s biological signal (A-to-G mismatch).

**Figure 3:**
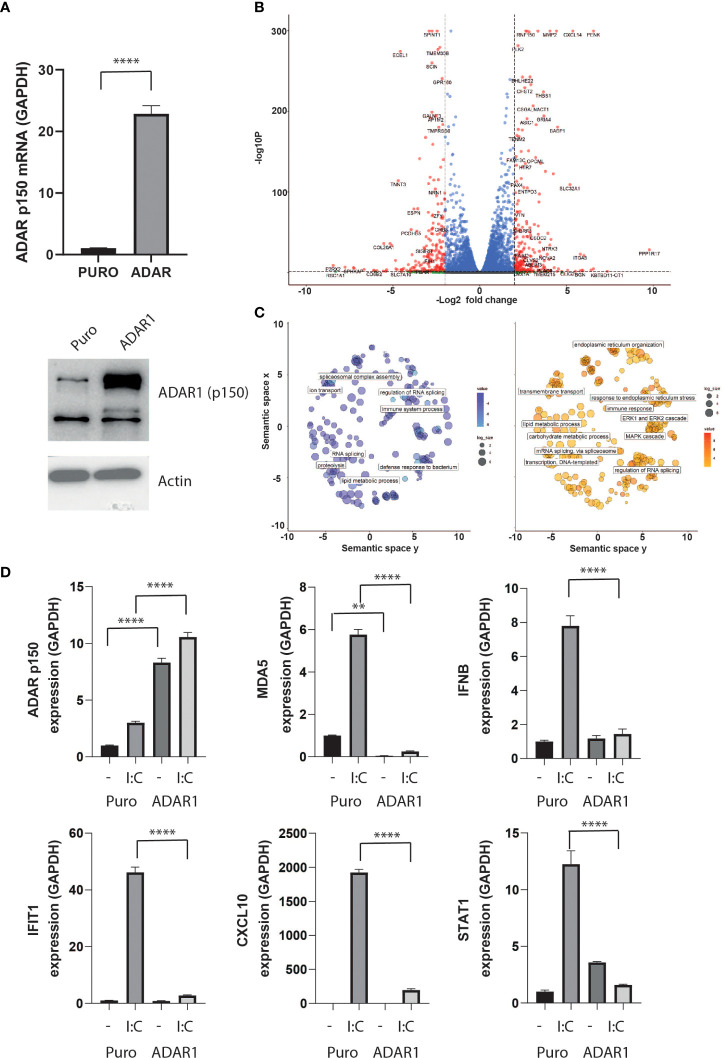
ADAR1 overexpression inhibits the type I IFN response. **(A)** ADAR1 p150 expression in EndoC-βH1 and EndoC- βH1 (ADAR1) cells determined by qPCR (upper panel) and western blot analysis using Anti-ADAR1 #ab168809, Abcam (lower panel). **(B)** Volcano plot on differential expressing genes after *ADAR1* overexpression. Dashed lines show log_2_FC ≤ -2 or log_2_FC ≥2 and adj. P value < 0.05. Plot was generated using Enhanced Volcano **(C)** Pathway analysis on downregulated (left, log_2_FC ≤ -2, adj. P value < 0.05) and upregulated (right, log_2_FC ≥2, adj. P value < 0.05) genes. Plots were generated using Revigo. **(D)** Gene expression of *ADAR1 p150, MDA5, IFNβ, IFITH1, CXCL10* and *STAT1* after ADAR1 overexpression in the presence or absence of polyI:C. N=3 independent experiments. Data are expressed as means of independent experiments ± SEM. Differences between groups were evaluated using one-way ANOVA or linear mixed model in case of missing values, followed by Bonferroni *post-hoc* test. **P<0.01 and ****P<0.0001.

Cells were lysed in RIPA buffer supplemented with protease inhibitor cocktail (Roche). Protein quantification was performed with the BCA protein assay kit (Thermo Fisher Scientific). 25 μg protein extracts were loaded on 10% acrylamide/bis acrylamide SDS page gel. After electrophoresis, protein transfer was performed onto a nitrocellulose membrane (GE Healthcare). Membranes were stained with primary antibodies overnight at 4°C and secondary HRP conjugated antibodies (Santa Cruz Biotechnology) for 1 hour RT. Antibodies used were: anti-ADAR1 [#ab168809, Abcam (**Figures 1**, **2**) and #14175, Cell Signaling Technology (**Figure 3**)], anti-STAT1 and anti-STAT2 (#14995 and #72604, Cell Signaling Technology) and as a loading control anti-actin (#0869100, MP Biomedicals) or anti-tubulin.

### Lentiviruses production and transduction

The vectors were produced as described previously (29). Viral supernatants (MOI=2) were added to fresh medium supplemented with 8 µg/mL Polybrene (Sigma-Aldrich), and the cells were incubated overnight. The next day, the medium was replaced with fresh medium. Transduction efficiency was analyzed 3–6 days after transduction.

### RNA interference

EndoC-βH1 cells were transfected overnight with 30 nmol/L of siRNA and cells were kept in culture for 48 hours. Transfection was performed using siRNA targeting ADAR (5’-TTCCGTTACCGCAGGGATCTA-3’; 1027416, Qiagen, Venlo, The Netherlands) using Lipofectamine RNAiMax (Invitrogen, Carlsbad, CA, USA). Allstars Negative Control siRNA (siCTL; Qiagen) was used as a negative control.

### Assessment of cell apoptosis

Cells were stained with the DNA-binding dyes propidium iodide (PI) and Hoechst 33342 (10 µg/ml, Sigma-Aldrich) to count apoptotic cells under a fluorescent microscope. In each experimental condition, a minimum of 500 cells were counted by two independent observers (one of them unaware of sample identity).

### Data and materials availability

Bulk RNA-seq data that were generated by this study is available on the Gene Expression Omnibus (GEO) database under the accession number GSE214851. Other datasets mentioned are available on GEO using accession numbers GSE133218, GSE137136, GSE148058 and GSE108413.

## Results

### Cytokines trigger increased expression of ADAR1 in β cells

IFNα and IFNγ play important roles in T1D pathogenesis, from initiation of autoimmunity (IFNα) to the more advanced β cell destruction process (IFNɣ) (30, 31). To identify key pathways involved during T1D development, we used RNA-seq datasets from human islets and EndoC-βH1 cells exposed to IFNα (24h) or IFNγ/IL1β (48h), and searched for common differentially regulated genes (32, 33). This resulted in the identification of 623 common genes in EndoC-βH1 cells and 577 common genes in primary human islets. As expected, gene ontology pathway analysis identified IFN signaling and genes involved in HLA class I antigen peptide processing and presentation, highlighting the importance of the islet microenvironment in triggering cytotoxic T lymphocyte (CTL)-mediated β cell destruction (**Figure 1A**). In addition to immune-related genes, we observed that both IFNα and IFNγ/IL1β stimulation led to a significant increase in expression of ADAR1 in EndoC-βH1 cells and primary human islets suggesting an increased RNA deamination rate in β cells during inflammation (**Figure 1B**). We confirmed the effect of the different cytokines on *ADAR1* mRNA and protein expression in EndoC-βH1 cells using *STAT1* expression as a control for treatment effectiveness (**Figures 1C, D**). Of note, the expression of the isoform p150 of ADAR1 protein was undetectable in the absence of cytokine stimulation.

### Enhanced A-to-I editing in β cells following IFNα and proinflammatory cytokine stimulation

To determine the consequences of the observed high ADAR1 expression following proinflammatory cytokine stimulation, and to decipher the RNA editome, we screened for A-to-G RNA mismatches (i.e., inosines present in the RNA are reverse transcribed into guanosines in cDNA and A-to-I editing is detected as A-to-G mismatches) by comparing reads in IFNα and IFNγ/IL1β-treated samples and non-treated samples against genomic reference (34, 35). Using the RNA editing index to measure the global rate of editing in *Alu* regions, we observed that IFNα and IFNγ/IL1β specifically triggered A-to-I RNA editing in β cells and primary human islet samples (**Figures 2A, B**).

### ADAR overexpression inhibits the antiviral response while ADAR silencing exacerbates the effects of IFNα in β cells

To model the effect of ADAR1-p150 independently of the pleiotropic effects of cytokines, we generated a stable ADAR1-overexpressing human β cell line, by lentivirus transduction. In these cells, we detected an over 20-fold increase in ADAR1-p150 gene expression, and confirmed the corresponding increase in protein level by western blot analysis (**Figure 3A**). While ADAR1 overexpression had no major impact on endogenous ADAR1, PDX1 and MAFA gene expression, we observed a slight but significant increased NKX6.1 and a 50% decreased insulin gene expression suggesting that ADAR1 may interfere with β cell function (**Supplementary Figure 1A** **)**. Differential gene expression analysis performed on high-depth RNA-seq revealed profound transcriptome changes following ADAR1 overexpression. In total, 2,851 genes were differentially expressed (1,477 up-regulated, 1,374 down-regulated - |Log_2_FC| > 0.58; P adj. P value < 0.05) (**Figure 3B** and **Supplementary Data S1**). Among them, we observed regulation of genes involved in immune system processes and defense to bacterium, confirming a role for ADAR1 in immune response (**Figure 3C**).

To validate this observation, we triggered the type I IFN response in β cells by mimicking viral infection *via* poly-I:C transfection (36). Poly-I:C transfection led to an increase in IFNβ expression and downstream IFN-stimulated genes (ISG) such as *MDA5*, *IFIT1*, *CXCL10* and *STAT1*, but ADAR1 overexpression completely abolished this antiviral response (**Figure 3D**).

**Figure 4:**
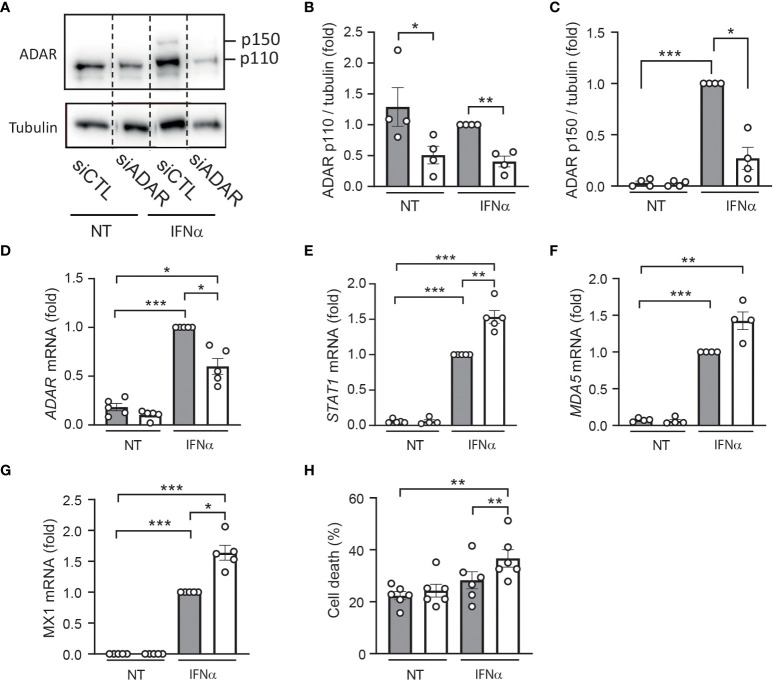
ADAR1 silencing exacerbates the effects of IFNα in human β cells. EndoC-βH1 cells were transfected with an siRNA control (siCTL: grey bars) or with an siRNA targeting ADAR (white bars) and left to recover for 48h. After this period, cells were left untreated (NT) or were treated with IFNα (2000 U/ml) for 24h. **(A)** Protein expression was measured by western blotting using Anti-ADAR1 antibody #14175 (Cell Signaling Technology) and representative images of 4 independent experiments are shown. Densitometry results are shown for ADAR p110 **(B)** and ADAR p150 **(C)**. mRNA expression of ADAR **(D)**, STAT1 **(E)**, MDA5 **(F)** and MX1 **(G)** was analyzed by RT-qPCR and normalized by β-actin. Values of siCTL + IFNα were considered as 1. **(H)** Cell death was evaluated using HO/PI staining. Data are expressed as means of independent experiments (shown as individual data points, n=4-6) ± SEM. Differences between groups were evaluated using one-way ANOVA or linear mixed model in case of missing values, followed by Bonferroni *post-hoc* test. *p<0.05, **p<0.01 and ****p<0.0001.

To confirm these data using a reverse approach, we used an siRNA targeting *ADAR1* leading to 40-70% reduction in gene and protein expression (**Figures 4A–D**). Of note, ADAR silencing had no effect on β cell identity genes and insulin expression (**Supplementary Figure 1B**). As expected, while IFNα treatment induced the expression of several ISGs [e.g., *STAT1* (**Figure 4E**) *MDA5* (**Figure 4F**) and MX1 (**Figure 4G**)], ADAR1 silencing potentiated the effect of IFNα on the expression of these antiviral genes and sensitized EndoC-βH1 cells to IFNα-mediated cell death (**Figure 4H**). Altogether, these data unveil a role for ADAR1 in dampening the type I IFN response to prevent an excessive inflammatory response potentially leading to β cell death.

### ADAR1 overexpression triggers alternative splicing events in β cells

**Figure 5:**
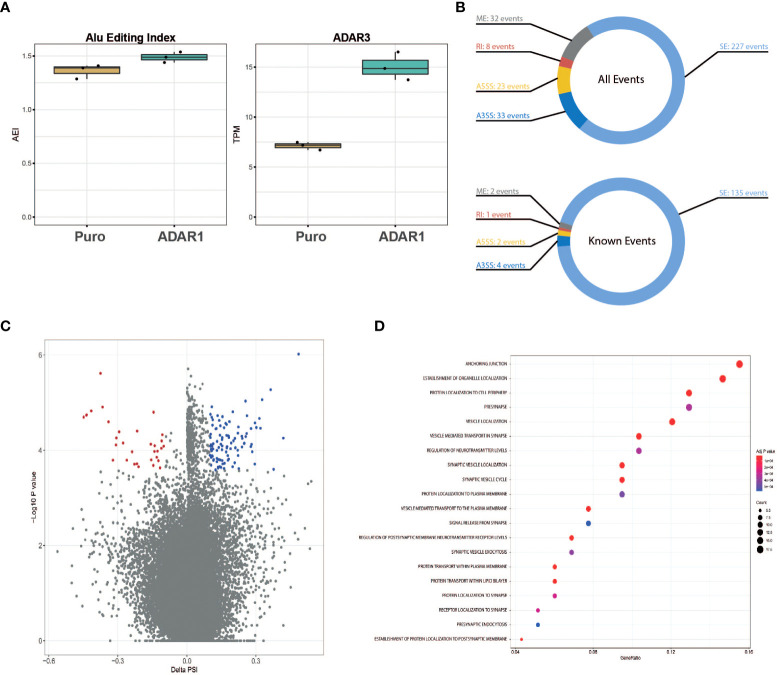
ADAR1 overexpression triggers alternative splicing in EndoC-βH1. **(A)** Global A-to-I RNA editing index across Alu elements (short interspersed nuclear elements) in Puro modified EndoC-βH1 and ADAR1 modified EndoC-βH1 cells (left panel), ADAR3 expression in Puro and ADAR1 modified EndoC-βH1. Data are shown as Transcript Per Million. **(B)** Donut charts representing the cumulated number of known and *de novo* alternative splicing events (top), and known events only (bottom). Events displayed have |ΔPSI| >0.10 and FDR <0.05. **(C)** Volcano plot of the inclusion and exclusion SE (skipped exon) events in EndoC-βh1 cells overexpressing ADAR1. Negative PSI indicates the inclusion of the event in ADAR1-overexpressing EndoC-βH1 cells whereas positive PSI indicates exclusion. Each dot represents an event with its ΔPSI (x-axis) associated to its *P*-value (y-axis). Colored dots (blue and red) represent genes with FDR<0.05. **(D)** Dot plot of the enrichment analysis, using Gene Ontology as reference, shows gene sets affected by known splicing events in cells overexpressing ADAR1. Gene ratio (x-axis) refers to the percentage of total genes input with alternative splicing events in selected GO terms (y-axis). SE, Spliced Exon; RI, Retain Intro; MXE, Mutually Exclusive Exon; A3SS, 3’ Alternative Splicing Site; A5SS, 5’ Alternative Splice Site.

Besides a role in immunity, gene ontology pathway analysis presented in **Figure 2C** revealed a possible role of ADAR1 in regulating alternative splicing. Considering the trend for an increased A-to-I *Alu* editing rate in ADAR1 overexpressing cells (**Figure 5A**), we studied the impact of adenosine deamination on the β cell coding transcriptome and searched for the presence of ADAR1-induced alternative splice variants. Of importance, in these cells, we observed an increased ADAR3 expression following ADAR1 transduction (**Figure 5A**).

After aligning the RNA sequencing reads to the reference genome, we identified a total of 323 alternatively spliced events (both known and *de novo*), modified by *ADAR1* overexpression (**Figures 5B, C** **)**. These events derived mainly from spliced exons (SE, 70%), but also mutually exclusive exons (ME, 10%) and alternative 3’ spliced sites (A3SS, 10%). Retained introns (RI, 7%) and alternative 5’ spliced sites (A5SS, 3%) were less abundant. Genes affected by alternative splicing were analyzed for pathway enrichment analysis using the REACTOME platform and were found to be mainly related to β cell function (e.g., pre-synapse, regulation of neurotransmitter levels), vesicle location (e.g., synaptic vesicle, vesicle-mediated transport to the plasma membrane) and protein transport (**Figure 5D**).

## Discussion

Our report positions ADAR1 as both an important player in dampening innate immunity in β cells and as a key editor of the β cell transcriptome. While exposure to inflammation, characteristic of the early or later stages of T1D development, is usually associated with deleterious effects, the data presented here recall earlier work on enhanced expression of Programmed death-ligand 1 PD-L1 detected in β cells from long-standing T1D individuals (37), suggesting that ADAR1, like PD-L1, is involved in the positive adaptive mechanisms to protect β cells from further destruction. During T1D, the induction of the unfolded protein response, following exposure to virus or inflammatory cytokines, participates in this adaptive phase to restore cellular homeostasis or to initiate apoptosis in the case of unresolved stress (38). At this decision point, the A-to-I editing induced by ADAR1 has been implicated in PERK activation and apoptosis induction *via* EIF2a/CHOP pathway (39). Other reports describe additional RNA-editing independent effects of ADAR1 *via* direct interaction with RIG-I, PKR or NF90 that could regulate cellular stress and the type I interferon response (3, 40, 41).

Supporting the concept that β cells are not passive victims in their destruction (8), our results show that the increased RNA editing rate correlates also with the emergence of novel transcript variants, demonstrating that ADAR1 activity is not only limited to *Alu* sequences but also affects coding regions with possible consequences for gene regulation and cell function (42). Surprisingly, ADAR1 p150 overexpression in EndoC-β H1 cells led a to concomitant increase in ADAR3 expression, which has been reported to act as a negative regulator of ADAR1-mediated editing (43). The competition between the different ADAR proteins may explain the relatively low editing rate measured in our samples and add to the complexity of gene editing regulation.

Despite the higher editing rate observed in inflammatory conditions or following ADAR1 overexpression, it is unlikely that all of the detected alternative splicing results solely from a direct A-to-I RNA editing of the target genes. As described here, ADAR1 overexpression led to extensive regulation of the RNA processing machinery or of spliceosome formation suggesting that ADAR1 may affect the transcriptome by modulating the expression of *trans*-regulatory elements. Among them, the splicing regulators ELAVL4 and NOVA2, previously reported as important splicing-regulatory RNA binding proteins involved in modulating β cell survival (44), were upregulated in response to ADAR1 transduction. The increased cell death observed after ADAR1-specific inhibition (**Figure 4**) is in line with this observation. Another report describes that the loss of RNA editing activity may lead to non-apoptotic cell death induction directly mediated by MDA5 (45), indicating that ADAR1 inhibition may lead to different forms of cell death. Of note, ADAR1 expression in our dataset led to decreased expression of pseudokinase mixed lineage kinase domain-like protein (MLKL) that serves as an effector in necroptosis.

The present results illustrate a central role of ADAR1 in β cells during inflammation and shed light on a novel regulatory mechanism potentially used by β cells to cope with environmental changes after viral infection but also during the different phases of inflammation. Although ADAR1-dependent effects are mostly protective, the functional and immunological consequences of mutations induced by RNA editing, including the potential generation of neoantigens, remain to be investigated.

## Data availability statement

The data presented in the study are deposited in the Gene Expression Omnibus (GEO) database under the accession number GSE214851. Other datasets mentioned are using accession numbers GSE133218, GSE137136, GSE148058 and GSE108413.

## Author contributions

FS, RC-F, ST and MC analyzed the RNA sequencing datasets and wrote the manuscript. ST and AB performed the experiments. AC and PM provided additional data for the revised manuscript. EL, DE and AZ supervised the project and wrote the manuscript. All authors contributed to the article and approved the submitted version.

## Funding

This research has been supported by the Israel Science Foundation (grant numbers 2039/20 and, 231/21 to EL), the DON Foundation and the Dutch Diabetes Research Foundation, JDRF and by IMI2-JU under grant agreement No 115797 (INNODIA) and No 945268 (INNODIA HARVEST). These joint undertakings receive support from the European Union’s Horizon 2020 research and innovation programme and European Federation of Pharmaceutical Industries and Associations (EFPIA), JDRF, and the Leona M. and Harry B. Helmsley Charitable Trust. DE acknowledges the support of grants from the Welbio-FNRS (Fonds National de la Recherche Scientifique) (WELBIO-CR-2019C-04), Belgium; the JDRF (3-SRA-2022-1201-S-B); the National Institutes of Health Human Islet Research Network Consortium on Beta Cell Death and Survival from Pancreatic β-Cell Gene Networks to Therapy [HIRN-CBDS]) (grant U01 DK127786). F.S. is supported by a Research Fellow (Aspirant) fellowship from the Fonds National de la Recherche Scientifique (FNRS, Belgium).

## Acknowledgments

The authors are grateful to Isabelle Millard, Anyishaï Musuaya, Nathalie Pachera and Cai Ying, (ULB Center for Diabetes Research), Steve Cramer and Martijn Rabelink (LUMC) for providing excellent technical support.

## Conflict of interest

The authors declare that the research was conducted in the absence of any commercial or financial relationships that could be construed as a potential conflict of interest.

## Publisher’s note

All claims expressed in this article are solely those of the authors and do not necessarily represent those of their affiliated organizations, or those of the publisher, the editors and the reviewers. Any product that may be evaluated in this article, or claim that may be made by its manufacturer, is not guaranteed or endorsed by the publisher.

## Supplementary material

Supplementary Figure 1 ADAR1 modulation and β cell identity and function. **(A)** ADARp150 and ADAR1 endogenous (upper panel), INS, PDX1, MAFA and NKX6.1 (lower panel) expression level following ADAR1 overexpression. **(B)** INS, PDX1, MAFA and NKX6.1 gene expression level upon ADAR1 specific inhibition by siRNA. Data are expressed as means of independent experiments (n=3) ± SD. Differences between groups were evaluated using unpaired t-test. ***p*<0.01 and *****p*<0.0001.

Supplementary Data Sheet 1 Differential gene expression detected in EndoC-βH1 following ADAR1 overexpression.

Supplementary Data Sheet 2 Gene-sets affected by alternative splicing in ADAR1 overexpressing cells.
