# Supplementary figures and images for: ADAR1-dependent editing regulates human β cell transcriptome diversity during inflammation
Source: Front Endocrinol (Lausanne). 2022 Nov 28;13:1058345. doi: 10.3389/fendo.2022.1058345 (PMC9742459; doi:10.3389/fendo.2022.1058345)

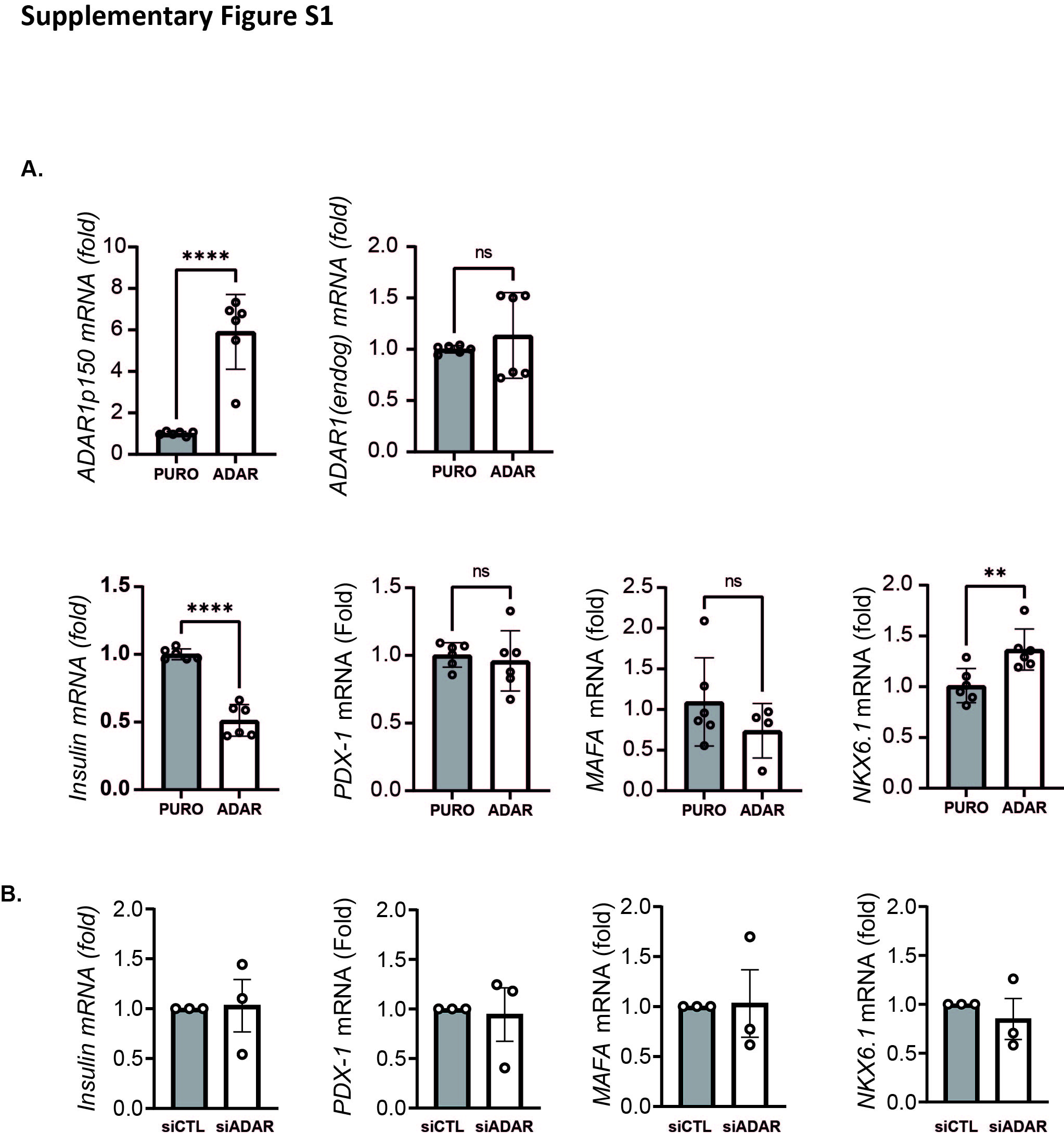

Supplement: Supplementary Figure 1 — ADAR1 modulation and β cell identity and function. (A) ADARp150 and ADAR1 endogenous (upper panel), INS, PDX1, MAFA and NKX6.1 (lower panel) expression level following ADAR1 overexpression. (B) INS, PDX1, MAFA and NKX6.1 gene expression level upon ADAR1 specific inhibition by siRNA. Data are expressed as means of independent experiments (n=3) ± SD. Differences between groups were evaluated using unpaired t-test. **p<0.01 and ****p<0.0001. [file Image_1.jpeg]
